# Traditional Chinese Medicine in Depression Treatment: From Molecules to Systems

**DOI:** 10.3389/fphar.2020.00586

**Published:** 2020-05-07

**Authors:** Chan Li, Junying Huang, Yung-Chi Cheng, Yuan-Wei Zhang

**Affiliations:** ^1^School of Life Sciences, Guangzhou University, Guangzhou, China; ^2^Department of Pharmacology, School of Medicine Yale University, New Haven, CT, United States

**Keywords:** traditional Chinese medicine, depression treatment, mechanism of action, systems pharmacology, neuropharmacology

## Abstract

Depression is a multigenetic or multifactorial syndrome. The central neuron system (CNS)-orientated, single target, and conventional antidepressants are insufficient and far from ideal. Traditional Chinese Medicine (TCM) has historically been used to treat depression up till today, particularly in Asia. Its holistic, multidrug, multitarget nature fits well with the therapeutic idea of systems medicine in depression treatment. Over the past two decades, although efforts have been made to understand TCM herbal antidepressants at the molecular level, many fundamental questions regarding their mechanisms of action remain to be addressed at the systems level in order to better understand the complicated herbal formulations in depression treatment. In this *Mini Review*, we review and discuss the mechanisms of action of herbal antidepressants and their acting targets in the pathological systems in the brain, such as monoamine neurotransmissions, hypothalamic–pituitary–adrenal (HPA) axis, neurotropic factor brain-derived neurotrophic factor (BDNF) cascade, and glutamate transmission. Some herbal molecules, constituents, and formulas are highlighted as examples to discuss their mechanisms of action and future directions for comprehensive researches at the systems level. Furthermore, we discuss pharmacological approaches to integrate the mechanism of action from the molecular level into the systems level for understanding of systems pharmacology of TCM formulations. Integration of the studies at the molecular level into the systems level not only represents a trend in TCM study but also promotes our understanding of the system-wide mechanism of action of herbal antidepressant formulations.

## Introduction

Depression is a chronic, prevalent, and debilitating mental illness that influences 15–20% of the population over the globe (Hasin et al., 2018). According to a recent report by the World Health Organization, depression is the leading cause of disability and a major contributor to the general burden of illness (WHO, 2017). Therefore, development of effective antidepressants will provide enormous social, economic, and health benefits.

Depression is not a unified syndrome, in which multiple underlying mechanisms exist. It is impossible to identify a specific factor that leads to or stops depression in all patients (Villas Boas et al., 2019). Hence, the better healing approach could be to seek the unique cause for each individual patient and then to apply a personalized treatment, not only for alleviating depression, but also for correcting the body's dysfunction that triggers depressive symptoms (Zhang and Cheng, 2019).

## TCM in Depression Treatment

The conventional antidepressants with single targets are insufficient and far from ideal. TCM has historically been used to treat depression up till today in clinical practice, particularly in Asia. TCM is a holistic medicine, which emphasizes the integrity of body and environmental effects on the internal homeostasis. In TCM, depression is thought to result from “vital energy” deficiency that is caused by dysfunction of multiple physiological systems in the body, such as dysregulation of blood circulation, inflammation, or “dampness and phlegm” (Ye et al., 2019). Strengthening “vital energy” is its healing principle, but correction of imbalance in other physiological systems by stimulating blood circulation, restraining inflammation, or removing “phlegm and dampness” is also needed (Feng et al., 2016).

It is well-known that a TCM herbal formula is more effective than single herbal molecules or herbs in clinical practice. There are numerous TCM herbal formulas, such as Kai-Xin-San (KXS), that have been usually used for depression treatment. Each formula is a mixture of multiple herbs that are proposed to act on diverse pathological targets simultaneously. Their composition and dosage rely on symptoms of individual patients. The holistic, multidrug, and multitarget nature of TCM fits well with the healing idea of systems medicine in the treatment of complex diseases, such as depression.

During the past two decades, efforts have been made to understand TCM in depression treatment; however, many fundamental questions regarding their mechanisms of action remain to be addressed. Previous studies have focused on revealing the mechanism of action at the molecular level by using either single herbal molecules or extracts from single herbs because the constituent complexity and drug–drug interactions of an entire formula often obstruct to uncover the molecular mechanism of action. However, it is vital to integrate the mechanism of action from the molecule level into the systems level in order to elucidate the system-wide mechanism of action of an herbal formula. The remarkable progress in our understanding of neurobiology of depression provides an opportunity to interpret the mechanism of action of herbal formulas at the systems level.

**Figure 1:**
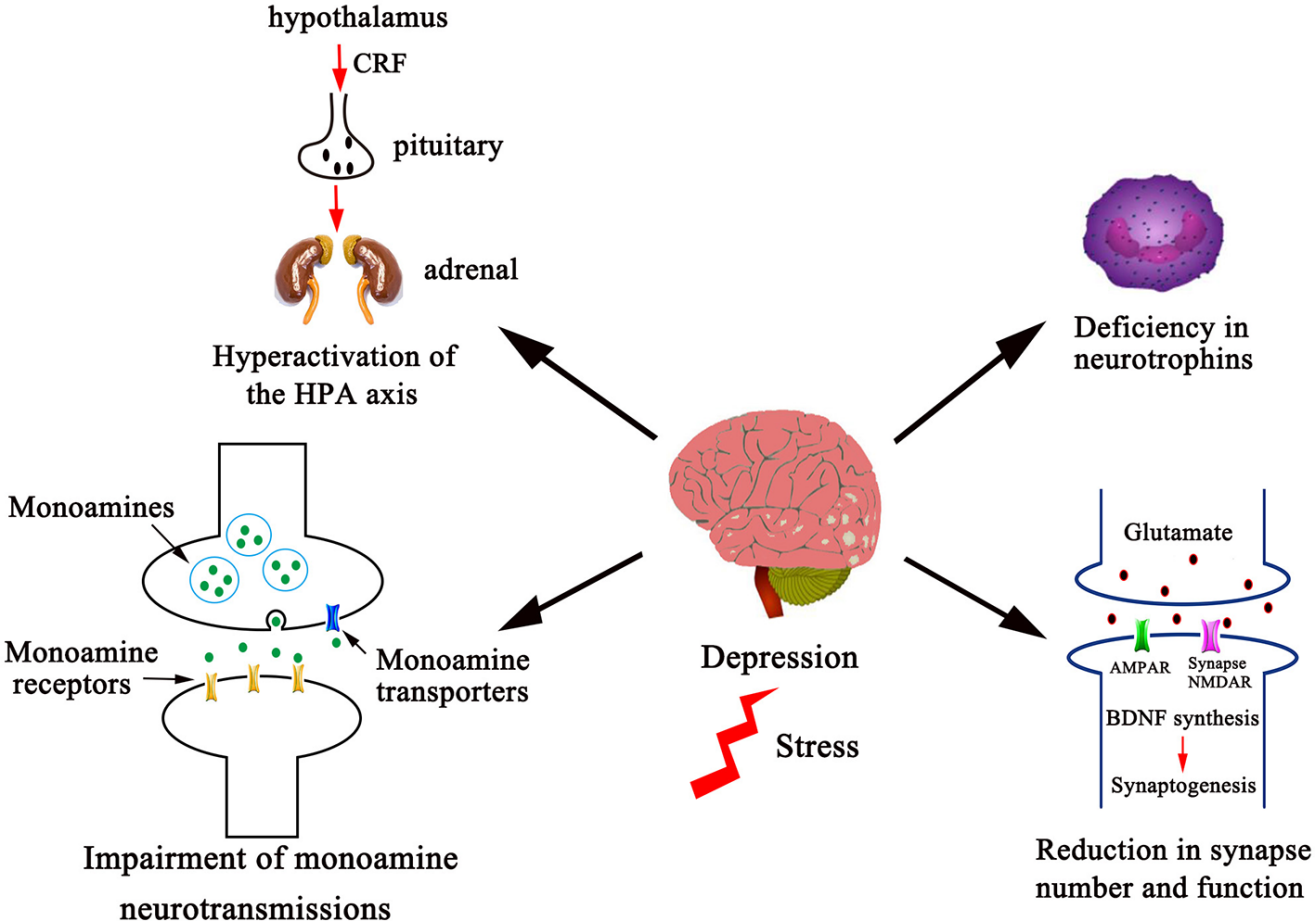
Several divergent systems in the CNS are involved in the pathophysiology of depression. The pathophysiological systems in the CNS that herbal antidepressants are proposed to act on include monoamine neurotransmissions, the HPA axis, neurotrophins, and synapse number and function as shown. Dysfunction of these systems leads to increased incidence of depression. Correspondingly, antidepressant discovery efforts toward these systems have provided numerous pharmacological targets, which include enhancement of monoaminergic transmissions, dehyperactivation of HPA axis, elevation of neurotrophic factor expression, and stimulation of glutamatergic transmission. Given space limitations, other factors in the pathophysiology of depression, such as the proinflammatory cytokines, the gastrointestinal system, ovarian steroids, vascular endothelial growth factor, and gene polymorphisms are not shown and discussed in this *Mini Review*.

Studies have revealed many divergent biological systems that are implicated in the pathophysiology of depression (Duman et al., 2016). These findings have provided numerous pharmacological targets that have been translated into the foundation to reveal the mechanism of action of antidepressants. In this *Mini Review*, we review and discuss the influences of herbal antidepressants on the pathological systems in the CNS (**Figure 1**), as well as the pharmacological approaches to integrate the mechanism of action of TCM antidepressant formulas from the molecular level into the systems level. A preliminary literature search for TCM herbal antidepressants was performed on the PubMed, Ovid, Google Scholar, and China National Knowledge Infrastructure and WanFang database by using keywords (Traditional Chinese medicine and depression or antidepressant and the term corresponding to the specific mechanism of action, such as monoamine transmission, HPA axis, BDNF, or rapid-acting antidepressant) without language restriction. Abstracts under the same mechanism of action category were independently screened by authors to identify articles of interest and full articles were further selected as representatives with a priority for single herbal molecules or herbs in the latest publications. Given space limitations, this review is not comprehensive; rather we give a few representative herbal molecules that are related to KXS (except for glutamate transmission) and well interpreted at the molecular level, and other herbal molecules are listed with a structural classification in **Table 1**.

**Table 1:** Herbal constituents that produce antidepressant-like activities in animal models or cells.

Structural category	Herbal constituents	Herbs	Mechanism of action	Models	Administration dosage	Treatment time	Reference
**Saponins**	Total saponins	*Panax ginseng* C. A. Mey.	The HPA axis/BDNF	CUMS rats	12.5, 25,50 mg/kg, i.g.	6 weeks	(Liu et al., 2011)
	Sarsasapogenin	*Anemarrhena asphodeloides* Bunge	Monoamine	CUMS mice	12.5, 25, 50 mg/kg, p.o.	14 days	(Ren et al., 2006)
	Ginsenoside Rb_1_	*Panax ginseng* C. A. Mey.	Monoamine	mice	4, 8, 16 mg/kg, p.o.	7 days	(Wang et al., 2017)
				mice	5, 10, 20 mg/kg, p.o.	60 min	(Wang et al., 2018b)
			BDNF	CUMS mice	20 mg/kg, p.o.	21 days	(Wang et al., 2019a)
	Ginsenoside Rg3	*Panax ginseng* C. A. Mey.	The HPA axis	CUS rats	20, 40 mg/kg, i.g.	14 days	(Xu et al., 2018)
			BDNF	CSDS mice	10, 20 mg/kg, i.p.	14 days	(You et al., 2017)
		*Panax notoginseng* (Burkill) F. H. Chen ex C. Chow & W. G. Huang	Glutamate transmission/BDNF	CMS mice	50, 100, 150 mg/kg, i.g.	4 weeks	(Zhang et al., 2017)
	Ginsenoside Rg1	*Panax ginseng* C. A. Mey.	BDNF	CUMS rats	40 mg/kg, i.p.	5 weeks	(Liu et al., 2016)
	Ginsenoside Rg5	*Panax ginseng* C. A. Mey.	BDNF	CSDS mice	5, 10, 20, 40 mg/kg, i.p.	14 days	(Xu et al., 2017a)
	Saikosaponin A	*Bupleurum chinense* DC.	Monoamine	CUMS rats	50 mg/kg, i.g.	4 weeks	(Guo et al., 2020)
	Saikosaponin D	*Bupleurum chinense* DC.	The HPA axis	CUMS rats	0.75 and 1.50 mg/kg, i.g.	21 days	(Li et al., 2017)
	YY-21	*Anemarrhena asphodeloides* Bunge	BDNF	CMS rats	10 mg/kg, i.g.	3 weeks	(Guo et al., 2016)
	YY-23	*Anemarrhena asphodeloides* Bunge	Glutamate transmission	CMS mice	20 mg/kg, i.g.	3 weeks	(Zhang et al., 2016)
	Yuanzhi-1	*Polygala tenuifolia* Willd.	Monoamine	CMS rats	2.5, 5, 10 mg/kg, p.o.	38 days	(Jin et al., 2015)
	YZ-50	*Polygala tenuifolia* Willd.	BDNF	CMS rats	140 and 280 mg/kg, i.g.	28 days	(Hu et al., 2010)
	Icariin	*Epimedium brevicornu* Maxim.	The HPA axis	SDM mice	25 and 50 mg/kg, i.g.	28 days	(Wu et al., 2011)
	20(*S*)‐protopanaxadiol	*Panax ginseng* C. A. Mey.	BDNF	CSDS mice	20 and 40 μmol/kg, i.p.	14 days	(Jiang et al., 2019)
**Glycosides**	Salidroside	*Rhodiola rosea* L.	The HPA axis/BDNF	Behavioral despair rats	20, 40 mg/kg, p.o.	14 days	(Yang et al., 2014)
	Total glycosides	*Paeonia lactiflora* Pall.	BDNF	CORT-induced rats	160 mg/kg, p.o.	21 days	(Mao et al., 2012)
	Gentiopicroside	*Gentiana lutea* L.	Glutamate transmission	Reserpine-induced mice	50, 100, 200 mg/kg, i.g.	3 days	(Liu et al., 2014b)
	Gastrodin	*Gastrodia elata* Bl.	BDNF	CUS rats	50, 100, 200 mg/kg, i.p.	14 days	(Zhang et al., 2014b)
	Paeoniflorin	*Paeonia lactiflora* Pall.	BDNF	CUMS mice	20 mg/kg, i.p.	30 days	(Liu et al., 2019)
**Flavonoids**	Flavonoid Extract	*Apocynum venetum* L.	BDNF	CORT-induced PC12 Cells	25, 50, 100 μg/ml	48 hours	(Zheng et al., 2011)
	Pueraria isoflavone	*Pueraria lobate* (Willd.) Ohwi	BDNF	Ovariectomy mice	10 and 100 mg/kg	8 weeks	(Tantipongpiradet et al., 2019)
	Puerarin	*Pueraria lobate* (Willd.) Ohwi	Monoamine/the HPA axis	CUS rats	60 and 120 mg/kg, i.g.	20 days	(Qiu et al., 2017)
			BDNF	Perimenopausal depression mice	30, 60, 120 mg/kg, i.g.	8 or 14 days	(Zhao et al., 2017)
	Curcumin	*Curcuma longa* L.	Monoamine/the HPA axis/BDNF	CUMS rats	2.5, 5 and 10 mg/kg, p.o.	21 days	(Xu et al., 2006)
				CUMS rats	40 mg/kg, i.p.	6 weeks	(Zhang et al., 2014a)
				WKY rats	50, 100, 200 mg/kg, i.p.	10 days	(Hurley et al., 2013)
	Genistein	*Glycine max* (L.) Merr.	Monoamine	Mice	5, 15, 45 mg/kg, p.o.	3 weeks	(Hu et al., 2017)
	Baicalein	*Scutellaria baicalensis* Georgi	BDNF	CMS rats	1, 2, 4 mg/kg, i.p.	21 days	(Xiong et al., 2011)
**Alkaloids**	Isorhynchophylline	*uncaria rhynchophylla* (Miq.) Miq. ex Havil.	Monoamine	Mice	10, 20, 40 mg/kg, i.g.	7 days	(Xian et al., 2017)
	Berberine Chloride	*Berberis aristata* Linn.	Monoamine	Male albino mice	5, 10, 20 mg/kg, i.p	15 days	(Kulkarni and Dhir, 2008)
	Piperine	*Piper Nigrum* L. *Piper longum* L.	BDNF	CORT-induced mice	5, 10 mg/kg, i.p.	21 days	(Mao et al., 2014)
	Tetrandrine	*Stephania tetrandra* S. Moore	Monoamine/BDNF	CUMS rats	10, 20, 40 mg/kg, i.g.	2 weeks	(Gao et al., 2013)
	Total alkaloid	*Aconitum carmichaelii* Debeaux	BDNF	Ovariectomized mice	10, 30 mg/kg, i.g.	7 days	(Liu et al., 2012)
	Scopolamine	*Solanaceae* Juss.	Glutamate transmission	*Gad1-Cre Camk2a-Cre* mice	25 μg/kg, i.p.	48 hours/3 times	(Wohleb et al., 2016)
	Huperzine A	*Huperzia* Bernh.	Monoamine/BDNF/Glutamate transmission	*CUMS rats*	0.05 and 0.15 mg/kg, i.g.	4 weeks	(Zheng et al., 2016; Du et al., 2017)
**Carbohydrates**	Oligosaccharide	*Morinda officinalis* How	The HPA axis	CORT-induced PC12 cells	5, 10, 125, 500 μM	5 days	(Li et al., 2003)
				CUS mice	12.5, 25, 50 mg/kg, i.g.	14 days	(Xu et al., 2017b)
	Fuzi polysaccharide 1	*Aconitum carmichaelii* Debeaux	BDNF	Mice	50, 100 mg/kg, i.p.	14 days	(Yan et al., 2010)
	Chiisanoside	*Acanthopanax* Miq.	BDNF	LPS-induced mice	2.5 and 5 mg/kg, i.p.	7 days	(Bian et al., 2018)
**Anthraquinones**	Emodi	*Rheum palmatum* L.	BDNF	CUMS mice	20, 40, 80 mg/kg, i.g	21 days	(Li et al., 2014)
**Terpenes and Phenylpropanoids**	Resveratrol	*Polygonum cuspidatum* Siebold et Zucc.	Monoamine	depression mice	30 mg/kg, p.o.	3 weeks	(Zhao et al., 2014)
			The HPA axis/BDNF	Mice	20, 40, 80 mg/kg, i.p.	21 days	(Wang et al., 2013)
			BDNF	LPS-induced mice/CUMS rats	80 mg/kg, i.p.	7 days/5 weeks	(Liu et al., 2014a; Ge et al., 2015)
	Trans-resveratrol	*polygonum cuspidatum* Siebold et Zucc.	Monoamine	CUS rats	40, 80 mg/kg, i.g.	21 days	(Yu et al., 2013)
	Rosmarinic acid	*Perilla frutescens* (L.) Britt.	BDNF	CUS rats	5 and 10 mg/kg, i.p.	14 days	(Jin et al., 2013)
	Crocin	*Crocus sativus* L.	BDNF	Rats	12.5, 25, 50 mg/kg, i.p.	21 days	(Vahdati Hassain et al., 2014)
	Cucurbitacin IIa	*Hemsleya amabilis* Diels	BDNF	CUMS mice	2.5, 5 mg/kg, i.p.	5 weeks	(Zhou et al., 2017)
	Hyperforin	*Hypericum perforatum* L.	BDNF	mice	4 mg/kg, i.p.	4 weeks	(Gibon et al., 2013)
			Glutamate transmission	Cortical neurons of rats	10 µM	9-12 days	(Kumar et al., 2006)
	Bakuchiol analogs	*Psoralea corylifolia* Linn.	Monoamine	Tr-CHO cells	0.03-333 µM	20 min	(Zhao et al., 2008)
	Honokiol	*Magnolia officinalis* Rehd. et Wils.	The HPA axis/BDNF	CUMS rats	2, 4, 8 mg/kg, i.g.	21 days	(Wang et al., 2018a)
	Macranthol	*Illicium dunnianum* Tutch.	BDNF	CUMS mice	10, 20, 40 mg/kg, p.o.	5 weeks	(Li et al., 2013)

Herbal constituents are listed according to their structural category and their sources, mechanism of action, animal models, and administration dosage and time are also given. Although TCM formulas have been shown to work more efficiently than single constituents or herbs in clinical practice, single molecules or herbs are often used for interpreting the mechanism of action at the molecular level due to the constituent complexity and drug–drug interactions of TCM formulas. For understanding the mechanism of action of a TCM formula at the systems level, it is essential to integrate the molecular mechanism into the system-wide mechanism of action. The representative TCM formulas in depression treatment are, but no limited to, Xiao-Yao-San (Chen et al., 2008), Kai-Xin-San (Fu et al., 2020), Jie-Yu-Wan (Feng et al., 2018), Shu-Yu-San (Chen et al., 2012), Chaihu-Jia-Longgu-Muli-Tang (Li et al., 2011), and so on. CUMS, chronic unpredictable mild stress; CSDS, chronic social defeat stress; CMS, chronic mild stress; SDM, social defeat model; CORT, corticosterone; CUS, chronic unpredictable stress; WKT, Wistar Kyoto; LPS, lipopolysaccharides.

## Molecular Mechanism of Action of TCM Herbal Antidepressants

### Monoamine Transmissions

In monoamine hypothesis, depression is caused by an impairment of monoamine neurotransmissions. Inhibition of monoamine reuptake transporters increases the availability of monoamines in the synaptic cleft and subsequently enhances monoamine transmissions. The monoamine reuptake transporters for serotonin (5-HT) and norepinephrine (NE) are the major targets for current available antidepressants. In addition, a host of proteins including monoamine metabolic enzymes and postsynaptic monoamine receptors are also involved in monoamine transmissions. The monoamine-based inhibitors enhance 5-HT or NE transmission, resulting in alterations in firing activity of dorsal raphe nucleus or locus coeruleus through different mechanisms (Mansari et al., 2010; Araragi et al., 2013).

It can be exemplified with the study on *Polygala tenuifolia* Willd., which has been shown to exert expectorant, tonic, tranquilizer and antipsychotic efficacies in clinical practice and can be seen in several empirical formulas for depression treatment, such as Kai-Xin-San (KXS) (Hu et al., 2011). Its mechanism of action in depression treatment had not been well understood until Yuanzhi-1, a triterpenoid saponin isolated from *Polygala tenuifolia* Willd., has recently been identified to be a triple monoamine reuptake inhibitor with a high potency (Jin et al., 2015). Moreover, Yuanzhi-1 and its several derivates have been shown to exert comparable antidepressant-like activities with the conventional antidepressant, duloxetine, in animal behavioral models (Jin et al., 2014). However, lack of selectivity for 5-HT or NE reuptake of these triterpenoid saponins increases our concerns about their addictive side effects caused by elevating synaptic concentrations of dopamine. Hence, it is interesting to know if there are some constituents that show antagonistic interactions with the triterpenoid saponins to normalize their effects on dopamine transmission in *Polygala tenuifolia* Willd.-containing formulas. In addition to its influence on monoaminergic systems, previous studies have shown that an oligosaccharide esters-enriched fraction YZ50 produces an antidepressant action in animal models through the HPA axis (Hu et al., 2010; Liu et al., 2010). These results indicate that *Polygala tenuifolia* Willd. possesses various antidepressant actions through multiple mechanisms. Therefore, further study to reveal the synergistic interactions between the constituents is required in order to integrate their effects on multiple biological systems into the system-wide mechanism of action of *Polygala tenuifolia* Willd.

Besides Yuanzhi-1 and its derivates, many other herbal molecules have also been shown to produce antidepressant-like activities through their impacts on monoamine transmission, although their pharmacological profiles have not been clearly revealed yet (**Table 1**). These compounds, ranging from polyphenols, saponins, alkaloids, and flavonoids, have no structural preference in their mechanisms of action underlying enhancement of monoamine transmissions.

### The HPA Axis

Stress leads to activation of the HPA axis usually reflected in high levels of glucocorticoids, which subsequently impair neuronal survival and neurogenesis and thereby result in depressive symptoms (Keller et al., 2017). It should be emphasized that the communications exist between the HPA axis and the CNS, endocrine, or immune system by neural, hormonal, or inflammatory interactions, and that these systems integrate into a network that underlies antidepressant action. For instance, monoamine-based antidepressants can not only reverse stress-induced hyperactivity of the HPA axis, but also attenuate the inflammatory changes by reducing the release of proinflammatory cytokines from activated microglia (Leonard, 2014; Ramirez and Sheridan, 2016; Simões et al., 2019). Similarly, agents that eliminate inflammatory effects also exert an antidepressant-like activity in animal models through the communication between the CNS and immune system (Zunszain et al., 2011). Furthermore, agents that directly target the HPA axis, such as glucocorticoid receptor antagonists, vasopressin receptor antagonists, and corticotropin-releasing hormone receptor antagonists, could also be effective antidepressants by blocking receptor activities to terminate the consequence of hormone secretions due to stress-induced hyperactivity of the HPA axis (Menke, 2019).

Ginsenoside Rg3, a protopanaxadiol ginsenoside from *Panax ginseng* C. A. Mey., has been recently reported to exert anxiolytic and antidepressant-like activities through dehyperactivation of the HPA axis by reducing corticotropin releasing hormone, corticosterone and adrenocorticotropic hormone in chronic unpredictable stress (CUS) animal models (Xu et al., 2018). Interestingly, a previous study demonstrated that this compound produces anti-inflammatory activities by reducing the level of inflammatory cytokines in the lipopolysaccharide-induced mice (Kang et al., 2017). This phenomenon of one herbal molecule with multiple functions is often seen in the study of herbal antidepressants, possibly due to either the crosstalk between the biological systems or nonspecific interactions with multiple systems.

Other herbal constituents have also been reported to produce antidepressant-like activities through their effects on the HPA axis (**Table 1**). However, all of these herbal constituents have not been clarified whether their effects are direct or indirect. We cannot exclude one scenario that herbal constituents could directly act on one biological system and then induce the responses from the HPA axis due to the cross-talk between these biological systems. Thus, it could obscure the pharmacological targets that herbal constituents actually act on and mislead us to understand their molecular mechanisms of action. Therefore, further studies are needed to clarify the contribution of herbal constituents to the system-wide antidepressant action.

### Neurotrophins

Brain-derived neurotrophic factor (BDNF) deficiency contributes to the pathophysiology of depression (Duman et al., 2019). Experimental observations have demonstrated that stress-induced downregulation of cAMP response element binding protein (CREB) mRNA level, and its phosphorylation, BDNF expression, and neurogenesis can be reversed by antidepressant treatments (Kishi et al., 2018). This raises the possibility that an agent that directly stimulates BDNF singling cascade might be an effective antidepressant. The potential drug targets in BDNF cascade should enhance CREB activity and BDNF expression, activate BDNF receptor TrkB, or stimulate post-receptor signaling cascades such as Ras-Raf-ERK, PI3K-Akt, and PLC*γ*.

Recent studies have demonstrated that chronic administration (40 mg/kg, 5 weeks) of ginsenoside Rg1, a protopanaxatriol type of ginsenoside, reverses behavioral abnormality and downregulation of the phosphorylation level of CREB and BDNF expression in the prefrontal cortex induced by chronic unpredictable mild stress (CUMS) in rats (Zhu et al., 2016a; Yu et al., 2018). In addition, ginsenoside Rg1 has also been shown to exert neuroprotective effects by suppressing inflammatory pathway activity, inhibiting neuronal apoptosis, and stimulating synaptic-related protein expression, such as CREB, BDNF, PSD-95, and synaptophysin (Fan et al., 2018). Ginsenoside Rg5, a protopanaxadiol ginsenoside, has been reported to exert an antidepressant-like activity by reversing the chronic social defeat-induced decrease in hippocampal BDNF expression and phosphorylation of TrkB (Xu et al., 2017a).

Several other herbal constituents have also been reported to produce antidepressant-like activities through BDNF signaling cascade (**Table 1**). It will be interesting to know if these herbal constituents directly act on BDNF signaling cascade and what targets they specifically interact with. Hence, more in-depth studies are required to address these questions, which are important for our understanding of their mechanistic details in order to further refine the use of these herbal antidepressants.

### Glutamate Transmission

Glutamate transmission has recently received the most attention in the development of rapid-acting antidepressant agents. These agents, such as NMDA receptor channel blockers and its positive allosteric modulators and acetylcholine muscarinic (AChM) receptor antagonists, enhance glutamate transmission, subsequently increase BDNF release and synapse function, thus rapidly reverse stress-induced synaptic abnormalities (Koike et al., 2011; Li et al., 2011; Burgdorf et al., 2013). Ketamine, a NMDA receptor antagonist, has been shown to produce rapid antidepressant actions (Diazgranados et al., 2010; Murrough et al., 2013), and its *S* (+) enantiomer, esketamine was approved in 2019 as the first rapid-acting antidepressant to treat severe depression.

The *Solanaceae* Juss. family of herbs such as *Datura metel* L.*, Hyoscyamus niger* L., and *Datura stramonium* L. contain psychedelic tropane alkaloids used for surgical anesthesia in ancient TCM practice. Scopolamine, a major tropane alkaloid isolated from these herbs, can readily cross the brain blood barrier into the CNS to inhibit AChM1 receptor (Klinkenberg and Blokland, 2010). Recent studies have demonstrated that a single dose of scopolamine (25 µg/kg) exerts rapid antidepressant actions within days in rats (Furey et al., 2010; Drevets et al., 2013). Its antidepressant actions have been revealed to be mediated through blockade of AChM1 receptor on GABA interneurons and subsequently to increase in glutamate transmission and function of spine synapse (Voleti et al., 2013; Wohleb et al., 2016; Fogaça et al., 2019).

In addition to scopolamine, two herbal formulas, Yueju pill and Chaihu-jia-Longgu-Muli-tang, have recently been reported to exert rapid-acting antidepressant-like activities in animal models. A dose of ethanol extracts from Yueju pill (3 g/kg) rapidly attenuated depressive-like behaviors, increased hippocampal BDNF expression, activated prefrontal Akt-mTOR signaling, and downregulated NR1 expression within days (Xue et al., 2013; Tang et al., 2015; Xia et al., 2016). Chaihu-jia-Longgu-Muli-tang (a single dose of 2.1 g/kg) has also been shown to produce a rapid antidepressant-like activity in olfactory bulbectomization mice through activation of Akt-mTOR signaling and normalization of AMPA receptor/NMDA receptor ratio in PFC (Wang et al., 2019b). In addition, these formulas have previously been shown to produce antidepressant actions through monoaminergic systems as well as the HPA axis in chronic animal models (Mizoguchi et al., 2003; Li et al., 2012; Wang et al., 2013). It is reasonable that these formulas possess multiple antidepressant actions through several underlying mechanisms due to its multidrug property, but further study is needed to reveal the synergistic interaction between its rapid antidepressant action and other underlying mechanisms.

## Transition from Molecules to Systems

Studies have revealed numerous pathological factors that are involved in the pathophysiology of depression (Krishnan and Nestler, 2008). In addition to the factors or systems in the CNS mentioned above, other notable factors include proinflammatory cytokines, ovarian steroids, gastrointestinal system and microbiome, and vascular endothelial growth factor (Schmidt et al., 2011). The CNS-orientated and single target antidepressants can only be used to alleviate depressive symptoms, but not to correct dysfunction of the pathological factors in other biological systems. On the other hand, the holistic and multidrug approach of TCM formulation is proposed to simultaneously act on multiple targets across various systems in the pathophysiology of depression. Therefore, it is essential to investigate the mechanism of action at the systems level for better understanding of TCM formulations in depression treatment.

It is a challenge to investigate the pharmacology of any TCM formulation at the systems level, including synergistic interaction and compatibility between herbs within multiherb combinations, due to the fact that not all potentially bioactive ingredients from any given TCM formula were identified and that their pharmacological properties were not thoroughly defined (Zhou et al., 2016). However, several studies have successfully been conducted to examine the synergistic, additive, and antagonistic interactions of herb pairs in complex TCM formulas (Adams et al., 2006; Yi and Wetzstein, 2011; Wang et al., 2012). KXS, a combination of four herbs (*Panax ginseng* C. A. Mey., *Polygala tenuifolia* Willd., *Acorus tatarinowii* Schott, and *Poria cocos* (Schw.) Wolf), is an empirical formula for depression treatment. A recent study has been performed to optimize the compatibility of herb pairs in KXS by examining the activation of neurofilament expression in PC12 cells (Yan et al., 2015). In this study, *Panax ginseng* C. A. Mey. and *Polygala tenuifolia* Willd. were placed as an herb pair with a function in invigorating “vital energy”, while *Acorus tatarinowii* Schott and *Poria cocos* (Schw.) Wolf were assigned to another pair to eliminate “dampness and phlegm”. The study showed that an optimized KXS with an herb pair ratio (1:5) produced the greatest capability in promoting the expression of neurofilament and that two herb pairs exert strong synergistic interactions in stimulating neuronal differentiation.

KXS has previously been demonstrated to exert antidepressant actions through multiple mechanisms across biological systems, including increase in monoamine availability (Zhou et al., 2012; Zhu et al., 2012), activation of hippocampal synaptogenesis and BDNF signaling cascade (Zhu et al., 2016b; Yan et al., 2016; Zhu et al., 2017), dehyperactivation of the HPA axis (Dang et al., 2009), and enhancement of lipid metabolism (Zhou et al., 2020). It is evident that the antidepressant efficiency of KXS results from the synergistic interactions between individual herbs, although each herb showed the potent effect in depression treatment (Yan et al., 2015). A recent study has been conducted to screen the proteins in response to KXS administration (0.6 g/kg, 14 days) across biological systems by using quantitation-based proteomics (Dong et al., 2020). In this study, total 33 proteins with altered expression levels were identified to be associated with KXS treatment. Functional analysis further revealed that these proteins are implicated in glutamate signaling, synaptic plasticity, metabolic process, cell survival process, and BDNF, mTORC1, and cAMP pathways. These studies indicated that KXS exerts antidepressant actions across multiple biological systems and provided pharmacological approaches to our understanding of the mechanism of action of KXS at the systems level.

## Discussion

The studies, in which the herbal constituents or single molecules were used for exploring the mechanism of action at the molecular level, have provided a foundation to understand the system-wide mechanism of action of an herbal formula. Systems pharmacology studies drugs, drug targets, and drug effects at the systems level and reveals all responses across various biological systems to the pharmacological action of drugs (Zhao and Iyengar, 2012). Therefore, application of systems pharmacology approaches to TCM study is vital for our understanding of the system-wide mechanism of action of herbal formulas.

Although single herbal molecules or herbs show the potent action in depression treatment, an herbal composite formula is used clinically, rather than a single form. The empirical formulas have been proven to have greater efficacy and safety than single drugs in clinical practice, possibly due to their synergistic interactions and mutual detoxification (Ung et al., 2007). The synergy of multiple herbs in an herbal formula could be triggered by the interactions between herbal molecules from different herbs or between the pharmacological targets across biological systems that herbal molecules specifically act on, and this phenomenon could be interpreted through systems pharmacology study of TCM herbal formulations.

We should acknowledge that a major challenge is lack of an integrated database including all interactions between the pathological factors across biological systems in the pathophysiology of depression, although efforts have been made to identify the specific interactions within the CNS (Pittenger and Duman, 2008). The effects of other biological systems on the CNS remain to be thoroughly studied in order to reveal the interactions between the pathological factors in different biological systems. With such a database, we will be able to promote our study from the molecular level into the systems level, which, in turn, could facilitate the integration of other biological systems with the CNS in depression treatment.

In summary, integration of the studies at the molecular level into the systems level not only represents a trend in TCM study but also promotes our understanding of the system-wide mechanism of action of herbal formulas. With many available techniques in systems biology, neurobiology, and pharmacology, the study of TCM will assist in developing future medications or approaches for systematic and effective depression treatment.

## Author Contributions

CL, JH, Y-CC, and Y-WZ wrote the manuscript.

## Conflict of Interest

The authors declare that the research was conducted in the absence of any commercial or financial relationships that could be construed as a potential conflict of interest.
